# Fission yeast cells deficient in siderophore biosynthesis require Str2 for ferrichrome-dependent growth

**DOI:** 10.3389/fmicb.2025.1527727

**Published:** 2025-02-06

**Authors:** Berthy Mbuya, Samuel Plante, Tobias Vahsen, Ariane Brault, Simon Labbé

**Affiliations:** Département de Biochimie et de Génomique Fonctionnelle, Faculté de médecine et des Sciences de la santé, Université de Sherbrooke, Sherbrooke, QC, Canada

**Keywords:** fission yeast, ferrichrome, iron, iron-regulatory GATA-type transcription factor, siderophore transporter

## Abstract

Ferrichrome (Fc) acquisition in *Schizosaccharomyces pombe* is mediated by the cell-surface siderophore-iron transporter Str1. Here, we report that Str2, a protein homologous to Str1, localizes to the vacuolar membrane. Like Str1, Str2 expression is transcriptionally regulated in response to changes in iron concentrations. Both the *str2^+^* and *str1^+^* genes are induced under low-iron conditions and are repressed by the iron-responsive GATA-type transcription factor Fep1 when iron is abundant. Under high-iron conditions, chromatin immunoprecipitation (ChIP) assays reveal that TAP-Fep1 occupies the *str2^+^* and *str1^+^* promoters. Isolated vacuoles from *str2Δ fep1Δ* cells expressing GFP-tagged Str2 exhibit iron accumulation in vacuoles upon exposure to exogenous holo-Fc. *sib1Δ sib2Δ* cells deficient in Fc biosynthesis and lacking the *str2^+^* gene (*str2Δ*) are unable to grow in the presence of exogenous Fc as a sole source of iron. Further analysis identified that conserved amino acids Tyr^539^ and Tyr^553^ in the last predicted loop of Str2 are required for supporting Fc-dependent growth of a *sib1Δ sib2Δ* mutant strain. Collectively, these findings indicate that the vacuolar Str2 protein plays a role in the consumption of Fc as an iron source, while also revealing the involvement of the vacuole in iron release from exogenous Fc after its assimilation.

## Introduction

The acquisition of the transition metal iron is essential for aerobic organisms. Due to its redox properties, iron serves as an indispensable cofactor in the catalytic centers of several enzymes, including those involved in the respiratory chain, energy production, amino acid biosynthesis, and the detoxification of reactive oxygen species (Puig et al., 2017; Galaris et al., 2019; Katsarou and Pantopoulos, 2020; Philpott et al., 2020). In aerobic environments, iron predominantly exists as ferric oxyhydroxides, which are poorly soluble and therefore not readily bioavailable (Aguiar et al., 2021). Given this limited iron availability, all organisms, including fungi, have evolved different mechanisms to mobilize and assimilate iron from their surroundings.

One strategy used by the fission yeast *Schizosaccharomyces pombe* is the synthesis, accumulation, and secretion of the hydroxamate-type siderophore ferrichrome (Fc) (Schrettl et al., 2004b; Haas et al., 2008; Mercier and Labbé, 2010; Plante and Labbé, 2019). The biosynthesis of Fc in *S. pombe* requires the Sib2, Sib3, and Sib1 proteins (Schrettl et al., 2004b; Brault et al., 2022). First, the ornithine-N^5^-oxygenase Sib2 catalyzes the N^5^ hydroxylation of the precursor ornithine. The resulting N^5^-hydroxyornithine is subsequently acylated by the N^5^-transacetylase Sib3, forming N^5^-acetyl-N^5^-hydroxyornithine. Finally, this compound, in combination with three glycine residues, is used to assemble Fc through the activity of the non-ribosomal peptide synthetase (NRPS) Sib1, a multimeric enzyme capable of generating peptide-based molecules (Schrettl et al., 2004a; Haas et al., 2008; Haas, 2014). Microscopic analyses of *S. pombe* cells expressing functional fluorescently tagged Sib1, Sib2, and Sib3 proteins have revealed that all three proteins share a common cytosolic subcellular localization under low-iron conditions (Brault et al., 2022). Furthermore, protein–protein interaction studies have shown that Sib2 and Sib3 are interacting partners when cells are cultured under iron-deficient conditions (Brault et al., 2022; Mbuya et al., 2024).

Once synthesized, a portion of Fc is excreted into the extracellular environment to capture ferric ions from various sources (Schrettl et al., 2004b). Despite the relatively low levels of extracellular Fc secreted by *S. pombe* (less than 5% of its total intracellular Fc content), this extracellular pool of Fc is sufficient to promote the growth of *Saccharomyces cerevisiae fet3Δ arn1-4Δ* cells expressing the Fc transporter Arn1, as well as *Aspergillus nidulans sidAΔ* cells, in cross-feeding co-culture assays (Schrettl et al., 2004b; Brault et al., 2022; Chiu et al., 2022). Fc-bound iron (holo-Fc) is subsequently retrieved by *S. pombe* cells via the cell-surface siderophore transporter Str1 (Pelletier et al., 2003; Plante and Labbé, 2019). Str1 is a member of the major facilitator superfamily (MFS) of transporters (Law et al., 2008). Studies have shown that heterologous expression of *S. pombe* Str1 complements the Fc assimilation deficiency of an *S. cerevisiae* mutant strain defective in holo-Fc uptake (Pelletier et al., 2003). Furthermore, experiments using an *S. pombe sib1Δ sib2Δ* strain, unable to synthesize Fc *de novo*, have shown that the Fc-dependent growth deficiency of this strain is rescued by Fc supplementation in the presence of a functional Str1 protein (Plante and Labbé, 2019).

The transcription of *sib1^+^*, *sib2^+^*, and *str1^+^* genes is differentially regulated in response to changes in iron concentrations. Expression of these genes is induced under iron-starvation conditions and repressed when iron is abundant (Plante and Labbé, 2019; Brault et al., 2022). The iron-dependent repression of *sib1^+^*, *sib2^+^*, and *str1^+^* is primarily governed by the iron-responsive transcription factor Fep1 (Pelletier et al., 2002; Pelletier et al., 2005; Plante and Labbé, 2019; Brault et al., 2022). Under iron-replete conditions, Fep1 interacts with its target genes by recognizing GATA-containing DNA sequences. In contrast, when cells undergo a transition from high to low iron concentrations, Fep1 loses its ability to bind to these GATA-binding elements *in vivo*, leading to the transcriptional induction of its target genes (Jbel et al., 2009).

The Genome Database for *S. pombe*, known as PomBase (Rutherford et al., 2024), indicates that Str1 has a paralog called Str2 (*SPCC61.01c*). Like Str1, Str2 is predicted to be a transmembrane protein, with an arrangement of transmembrane spans and hydrophilic loop regions that is predicted to form a tridimensional structure closely related to members of the major facilitator superfamily (MFS) of transporters (Law et al., 2008). The amino acid sequence identity between Str1 and Str2 is 29.0%, whereas their amino acid sequences show 48.8% similarity. Consistently, the predicted topological structures of these two transmembrane proteins exhibit a high degree of resemblance (Pelletier et al., 2003). However, the physiological role of Str2 in *S. pombe* remains unclear.

In this study, we determined that *str2^+^* mRNA levels are increased in iron-starved cells, whereas under basal and iron-replete conditions, mRNA levels for this gene are down-regulated. Chromatin immunoprecipitation assays showed that Fep1 is recruited to the *str2^+^* promoter in response to iron. Microscopic analysis revealed that a functional GFP-tagged Str2 localizes to the vacuole membrane in iron-deprived cells or in cells lacking Fep1. Purified vacuoles from *str2Δ fep1Δ* mutant cells exhibit reduced vacuolar iron content. Under low-iron conditions, *str2Δ sib1Δ sib2Δ* mutant cells deficient in Fc biosynthesis fail to grow when exogenous holo-Fc is used as the sole source of iron. Together, these results provide the first example of a resident vacuolar membrane-localized siderophore-iron transporter involved in Fc mobilization, which strengthens fission yeast cells against iron starvation.

## Materials and methods

### Strains, media, and growth conditions

The genotypes of *S. pombe* strains are listed in Table 1. Alleles were inactivated using a resistance gene cassette engineered for multiple use in fission yeast (Iwaki and Takegawa, 2004). This cassette contains the kanamycin/G418 resistance gene (*kanMX*) flanked by loxP sequences on either side. Each disruption cassette is flanked by short DNA segments homologous to the chromosomal sequences lying upstream and downstream of the gene to be deleted. After gene disruption, the cassette can be recycled by excision from the yeast genome using a Cre recombinase/loxP-mediated removal process (Gueldener et al., 2002). Yeast extract with supplements (YES) medium was used to grow yeast strains under standard conditions (Sabatinos and Forsburg, 2010). Edinburgh minimal medium (EMM) lacking specific nutrients was used to select transformed yeast strains carrying integrative or episomal plasmids.

**Table 1:** Yeast strains used in this study.

*S. pombe* strain	Genotype	Source
FY435	*h^+^ his7-366 leu1-32 ura4-*∆*18 ade6-M210*	Pelletier et al. (2002)
*fep1*Δ	*h^+^ his7-366 leu1-32 ura4-*∆*18 ade6-M210 fep1*∆*::ura4^+^*	Pelletier et al. (2002)
*fep1Δ php4Δ*	*h^+^ his7-366 leu1-32 ura4-∆18 ade6-M210 php4∆ fep1Δ::KAN^r^*	Jbel et al. (2009)
AMY58	*h^+^ his7-366 leu1-32 ura4-*∆*18 ade6-M210 sib1*∆ *sib2Δ::KAN^r^*	Mercier and Labbé (2010)
BMY2	*h^+^ his7-366 leu1-32 ura4-*∆*18 ade6-M210 sib1∆ sib2Δ fep1Δ::KAN^r^*	This study
BMY3	*h^+^ his7-366 leu1-32 ura4-*∆*18 ade6-M210 str2*∆*::KAN^r^*	This study
BMY4	*h^+^ his7-366 leu1-32 ura4-*∆*18 ade6-M210 str2*∆ *fep1∆::KAN^r^*	This study
BMY5	*h^+^ his7-366 leu1-32 ura4-*∆*18 ade6-M210 sib1∆ sib2Δ str2Δ::KAN^r^*	This study
BMY6	*h^+^ his7-366 leu1-32 ura4-*∆*18 ade6-M210 str2∆ fep1∆ abc3∆::KAN^r^*	This study
BMY7	*h^+^ his7-366 leu1-32 ura4-∆18 ade6-M210 sib1∆ sib2Δ str2Δ fep1Δ::KAN^r^*	This study
BMY8	*h^+^ his7-366 leu1-32 ura4-∆18 ade6-M210 abc3Δ fep1Δ::KANr*	This study
*fep1Δ cox4Δ*	*h^+^ his7-366 leu1-32 ura4-∆18 ade6-M210 cox4Δ fep1Δ::KANr*	This study

For transcript and protein steady-state level assessments, yeast liquid cultures were seeded to an OD_600_ of 0.5. When the cultures reached an OD_600_ of 1.0, cells were either left untreated or treated with the iron chelator 2,2′-dipyridyl (Dip, 250 μM) or FeCl_3_ (100 μM) for 1.5 h or 3 h, unless otherwise stated. For vacuole purification, the indicated cultures were treated with Dip for 3 h, with holo-Fc (1 μM) added during the final hour of treatment. Growth assays on solid media were performed by growing cells in YES medium to an OD_600_ of 1.0, then spotting serial dilutions (6,000 cells/10 μL; 600 cells/10 μL; and, 60 cells/10 μL) onto media without Dip or Fc supplementation (control) or supplemented with Dip (140 μM) or a combination of Dip and Fc (0.1 or 1 μM).

### Plasmids

To create the integrative plasmid pJK-1000*str2^+^-GFP*, a 2,791-bp EcoRI-BamHI PCR-amplified DNA fragment containing the *str2^+^* allele and its promoter region (starting from 1,000 bp upstream the initiator codon to the penultimate codon) was cloned into the EcoRI and BamHI sites of pJK148 (Keeney and Boeke, 1994). This plasmid was named pJK-1000*str2^+^nostop*. The GFP coding sequence was then amplified by PCR using primers designed to introduce BamHI and NotI restriction sites at the 5′ and 3′ ends, respectively. The resulting BamHI-NotI PCR-amplified DNA fragment was inserted in-frame with *str2^+^* at the corresponding sites in pJK-1000*str2^+^nostop*, generating the plasmid pJK-1000*str2^+^-GFP*. This plasmid was subsequently used to introduce mutations into the *str2^+^* coding sequence. In the *str2Y539A/Y553A* mutant allele, the codons for tyrosine (Tyr) at positions 539 and 553 were replaced by nucleotide triplets encoding alanine residues. For the *str2Y539A/R546A/Y553A* mutant allele, the same substitutions as in *str2Y539A/Y553A* were made, with an additional mutation replacing the arginine (Arg) codon at position 546 with a codon for alanine. These site-specific substitutions were introduced using a PCR overlap extension method (Ho et al., 1989). The construction of the plasmids pJK-1478*fep1^+^*, pJK-1478*TAPfep1^+^*, and pSP-808*abc3^+^-GFP* has been described previously (Pelletier et al., 2005; Pouliot et al., 2010).

### RNA extraction and mRNA expression analysis

Total RNA was extracted from cell cultures using the hot phenol method, as previously described (Chen et al., 2003). To analyze the transcript levels of *str2^+^* and *str1^+^*, real-time quantitative reverse transcription PCR (RT-qPCR) assays were performed, as previously described (Brault et al., 2022). The procedures for RT reactions, cDNA synthesis, and qPCR reactions were carried out as previously reported (Brault et al., 2022). Each target transcript was analyzed in experiments with a minimum of three biological replicates, with each sample reaction performed in triplicate. Results were considered valid if the target-specific fluorescent signal produced a C_t_ value ≤37 cycles, and all positive and negative control reactions yielded successful amplification and no amplification, respectively. Fold changes in *str2^+^* or *str1^+^* transcript levels between wild-type and *fep1Δ* mutant samples were calculated using the ΔΔCt method, normalized to the internal control *act1^+^* (Livak and Schmittgen, 2001; Schmittgen and Livak, 2008; Protacio et al., 2022). The following equation was used for calculations: ΔΔCt = [(Ct gene–Ct ref) in wild-type] versus [(Ct gene–Ct ref) in *fep1Δ*]; or, ΔΔCt = [(Ct gene–Ct ref) in *sib1Δ sib2Δ*] versus [(Ct gene–Ct ref) in *sib1Δ sib2Δ fep1Δ*], under the indicated experimental conditions related to iron availability. In the case of *str2^+^*, the primer pair allowed the detection of an amplicon corresponding to the coding region between positions +502 and + 601 down to the first nucleotide of the initiator codon. For *str1^+^*, the amplicon covered the coding region from positions +376 to +475, down to the A of the start codon. To detect *act1^+^* expression, a primer pair was used to amplify the coding sequence between positions +173 and + 280 down to the first base of the ATG codon of *act1^+^*.

### ChIP assays

Early logarithmic *fep1Δ php4Δ* cells expressing untagged or TAP-tagged *fep1^+^* alleles were grown in the presence of FeCl_3_ (Fe, 75 μM). When cultures reached an OD_600_ of 0.5, they were washed and incubated with either Dip (250 μM) or FeCl_3_ (Fe, 100 μM) for 3 h. Following the treatments, *in vivo* chemical cross-linking of proteins was performed by incubating the cell cultures in the presence of 1% formaldehyde for 20 min. The crosslinking reaction was neutralized by adding glycine (0.45 M), and cell lysates were prepared by glass bead disruption, as previously described (Larochelle et al., 2012; Brault et al., 2016). The samples were subsequently sonicated to shear the chromatin DNA into fragments of 500 to 1,000 bp. Immunoprecipitation of TAP-tagged Fep1 bound to chromatin was conducted using immunoglobulin G (IgG)-Sepharose beads, following the procedures as previously described (Jacques et al., 2014). Bead manipulation, which includes washings, elution, reversal cross-linking, and DNA precipitation, was performed in accordance with the protocols previously described (Adam et al., 2001; Jbel et al., 2009). Quantification of the immunoprecipitated DNA was conducted by quantitative real-time PCR (qPCR) using different sets of primers targeting the promoter regions of *str2^+^* and *str1^+^*. The occupancy of TAP-Fep1 at the *str2^+^* or *str1^+^* loci was determined by calculating the enrichment of the specific *str2^+^* and *str1^+^* promoter regions relative to a GATA-free 18S ribosomal DNA coding region, which served as an internal background control. The primer pairs used for amplifying the *str2^+^* and *str1^+^* promoter regions were: str2-105 (5’-CCAACTTCATTAAACATCTCGGTTAG-3′)/str2-2 (5’-CAGAGTGTATGGTAAATGGCAGTA-3′), and str1-921 (5’-GACAGTCCCGTACAAGGAAGAA-3′)/str1-812 (5’-AAGATGGAGGTGAAGGCAACTT-3′), respectively. The primers used for amplifying the 18S ribosomal DNA coding region were described previously (Brault et al., 2016). Each qPCR reaction was performed in triplicate using the Perfecta SYBR Green Fast mix (Quanta) on a CFX96 Touch Real-Time PCR instrument (BioRad). All ChIP experiments were repeated at least three times with independent chromatin preparations.

### Protein extraction and fluorescence microscopy

For all growth conditions, phenylmethylsulfonylfluoride (PMSF) (1 mM) was added directly to the cell cultures 15 min before harvesting to protect proteins from proteolysis. Whole cell extracts were prepared with glass beads using a FastPrep-24 instrument (MP Biomedicals, Solon, OH). Cells were lysed in HEGN_100_ buffer containing 20 mM 4-(2-hydroxyethyl)-1-piperazineethanesulfonic acid (HEPES), pH 7.9, 100 mM NaCl, 1 mM ethylenediaminetetraacetic acid (EDTA), 10% glycerol, 0.1 mM Na_3_VO_4_, 1 mM PMSF, 1 mM dithiothreitol (DTT), and a complete protease inhibitor mixture (Sigma-Aldrich; P8340). Cell lysates were incubated in the presence of Triton X-100 (1%) for 30 min at 4°C. Equal concentrations of each sample were resuspended in loading buffer (100 mM Tris–HCl, pH 7.5, 1.4 M *β*-mercaptoethanol, 140 mM sodium dodecyl sulfate (SDS), 5 mM EDTA, 4 M urea, 1 M thiourea, and 0.72 mM bromophenol blue), and proteins were resolved by electrophoresis on 6% SDS-polyacrylamide gels. Proteins were then transferred to nitrocellulose membranes, and the following antibodies were used for immunodetection of Str2-GFP, Abc3-GFP, and *α*-tubulin: monoclonal anti-GFP antibody B-2 (Santa Cruz Biotechnology) and monoclonal anti-α-tubulin antibody B-5-1-2. After incubation, the membranes were washed and incubated with appropriate horseradish peroxidase-conjugated secondary antibodies, developed with enhanced chemiluminescence (ECL) reagents (Amersham Biosciences), and visualized using an ImageQuant LAS 4000 instrument (GE Healthcare).

Cells were subjected to microscopic analysis using 1,000× magnification using the following filters: 340–380 nm (bimane-GS), 465–495 nm (GFP-tagged proteins), and 510–560 nm (FM4-64). Both fluorescence and differential interference contrast (DIC) images (Nomarski) were captured using a Nikon Eclipse E800 epifluorescence microscope equipped with a Hamamatsu ORCA-ER digital cooled camera. The representative fields shown in the images were obtained from a minimum of three independent experiments. Furthermore, the cell fields shown represent protein localization in 200 cells tested per condition.

### Isolation of intact vacuoles and bathophenanthrolinedisulfonic acid (BPS)-based spectrophotometric assay

The indicated strains were subjected to cell wall digestion, cell-surface membrane disruption, and then cell fractionation using a differential centrifugation method as previously described (Sooksa-Nguan et al., 2009). After the second Percoll step gradient (50%, v/v), the integrity of the isolated vacuoles was assessed by monitoring their ability to actively retain the fluorescent compound bimane-GS. This fluorescent compound is derived from the nonfluorescent, membrane-permeant monochlorobimane. Upon entering the cells, monochlorobimane is glutathionylated by cellular glutathione S-transferases, resulting in the production of bimane-GS, which is actively transported into the vacuoles, where it becomes fluorescent (Sarry et al., 2007).

Vacuole preparations were used either to quantify iron content or to detect the presence of GFP-tagged Str2 and Abc3 proteins. For protein detection, purified vacuoles were disrupted using glass beads in Thorner buffer (40 mM Tris–HCl, pH 6.8, 0.1 mM EDTA, 5% SDS and 8 M urea) supplemented with 1% Triton X-100. Vacuole lysates were incubated in a thermomixer at 37°C for 15 min with periodic rotation at 600 rpm, followed by an additional round of glass bead disruption using a MP-24 FastPrep instrument. The extracted vacuolar proteins were then subjected to immunoblot assays to detect Str2-GFP and Abc3-GFP.

For measuring vacuole iron content, the vacuole preparations were washed and lysed in a Tris–HCl solution (50 mM, pH 7.5) supplemented with Triton X-100 (1%), using glass beads disruption. The protein concentrations of the vacuolar lysates were determined using the Bradford assay, and equal amounts of these extracts were treated with citric acid (100 mM, pH 2.0) and incubated at 60°C for 4 h, as previously described (Rad et al., 2007). After centrifugation, the supernatant was mixed with an equivalent volume of citric acid and transferred to a fresh tube. BPS (5 mM) and freshly prepared ascorbic acid (100 mM) were then added. After 45 min of incubation at 25°C in the dark, the absorbance of the samples containing iron was measured, and the total iron content was calculated using a separate calibration curve, as previously described (Rad et al., 2007).

## Results

### Str2 expression is repressed in a Fep1-dependent manner under iron-replete conditions

**Figure 1:**
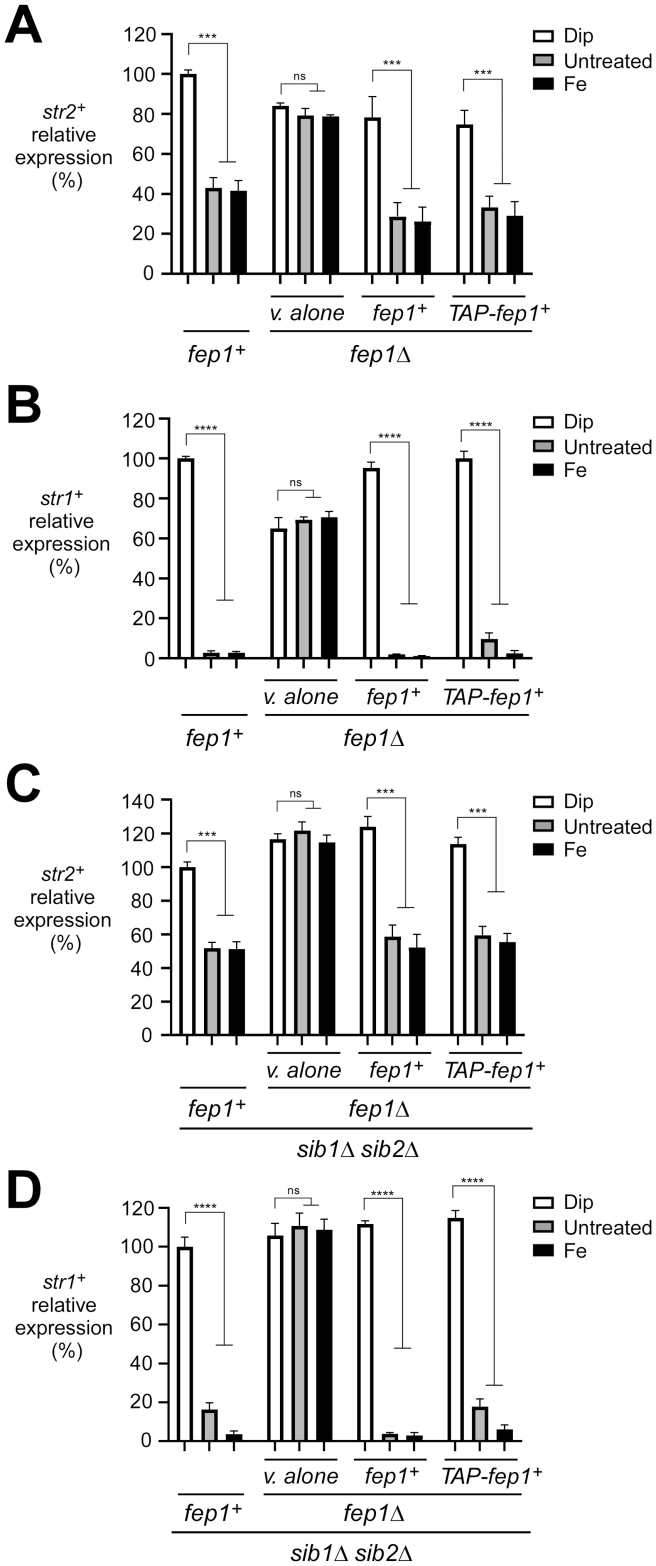
Assessment of the *str2^+^* and *str1^+^* transcript levels in response to iron availability. **(A–D)** Representative expression profiles of the *str2^+^* and *str1^+^* mRNAs in wild-type (*fep1^+^*), *fep1Δ*, *sib1Δ sib2Δ*, and *fep1Δ sib1Δ sib2Δ* strains. The cells were grown in YES medium to an OD_600_ of 1.0, followed by treatment with either Dip (250 μM) or FeCl_3_ (Fe, 100 μM) for 90 min, or left untreated. For the *fep1Δ* or *fep1Δ sib1Δ sib2Δ* strains, cells were transformed with either an empty integrative plasmid (v. alone) or a plasmid containing an untagged *fep1^+^* or TAP-tagged *fep1^+^* allele. After RNA isolation, the steady-state mRNA levels of *str2^+^* and *str1^+^* were analyzed by RT-qPCR assays. The graphs represent the quantification from three independent RT-qPCR experiments, with error bars indicating standard deviation (± SD; error bars). Statistical significance is represented by asterisks: *p* < 0.001 (***) and *p* < 0.0001 (****) (two-way ANOVA with Tukey’s multiple comparisons test, comparing the indicated strains grown under low-iron conditions), whereas “ns” denotes no significant difference. *str1^+^* was analyzed as a control gene, known to be repressed by iron.

Although previous studies have identified target genes of Fep1 (Pelletier et al., 2003; Rustici et al., 2007), several remain poorly characterized in terms of their roles in iron homeostasis, including the *str2^+^*-encoded putative siderophore transporter. To validate that *str2^+^* expression is repressed by iron repletion, we monitored *str2^+^* transcript levels in wild-type or *sib1Δ sib2Δ* strains either left untreated or treated with the iron chelator 2,2′-dipyridyl (Dip, 250 μM) or FeCl_3_ (Fe, 100 μM) for 90 min. As a control, we concurrently analyzed *str1^+^* transcripts, which are known to be down-regulated under iron-replete conditions (Pelletier et al., 2003; Plante and Labbé, 2019). For both strains, results showed that *str2^+^* and *str1^+^* transcript levels were highly expressed in the presence of Dip (Figures 1A–D). In contrast, their expression markedly decreased under basal and iron-replete conditions. In the wild-type strain, *str2^+^* and *str1^+^* mRNA levels were repressed 2.4- and 37.0-fold, respectively, in response to iron compared to levels under iron-starved conditions (Figures 1A,B). Similarly, in the *sib1Δ sib2Δ* mutant strain, *str2^+^* and *str1^+^* transcript levels were repressed 2.0- and 27.0-fold, respectively, by iron repletion compared to levels under low-iron conditions (Figures 1C,D). To examine the role of Fep1 in *str2^+^* gene regulation, similar experiments were performed using isogenic *fep1Δ* and *sib1Δ sib2Δ fep1Δ* strains. In both strains lacking Fep1, *str2^+^* and *str1^+^* transcripts exhibited high and constitutive expression levels that were unresponsive to iron for repression (Figures 1A–D). However, when the wild-type *fep1^+^* allele or a functional TAP-tagged *fep1^+^* allele was reintroduced by integration into *fep1Δ* and *sib1Δ sib2Δ fep1Δ* strains, the ability to repress *str2^+^* and *str1^+^* gene expression under basal and iron-replete conditions was restored (Figures 1C,D). Collectively, these results demonstrated that *str2^+^* is an iron-regulated gene, with its expression moderately repressed by Fep1 in response to iron.

### Under iron-replete conditions, Fep1 associates with *str2^+^* and *str1^+^* promoters *in vivo*

**Figure 2:**
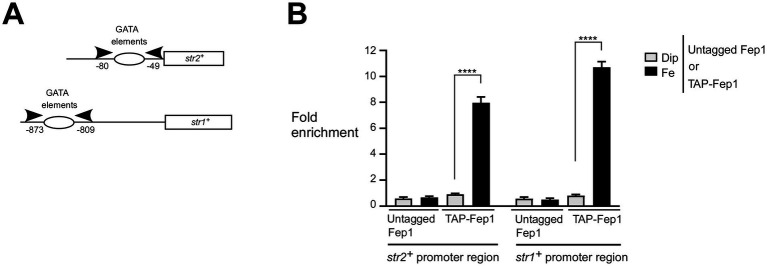
Fep1 binds to *str2^+^* and *str1^+^* promoters under iron-replete conditions. **(A)** Schematic representation of the *str2^+^* and *str1^+^* promoter regions. Arrowheads show primer positions for qPCR analysis. Nucleotide numbers correspond to the positions of primer binding relative to the A of the initiator codon of the *str2^+^* or *str1^+^* gene. Empty ovals depict promoter regions containing GATA elements, which are known to serve as binding sites for Fep1. **(B)** Logarithmic phase *fep1Δ php4Δ* cells expressing untagged or TAP-tagged *fep1^+^* alleles were incubated in the presence of Dip (250 μM) or FeCl_3_ (Fe, 100 μM) for 3 h. Following chromatin preparation and immunoprecipitation using Sepharose-bound anti-mouse IgG antibodies, specific regions of the *str2^+^* and *str1^+^* promoters were analyzed by qPCR to assess TAP-Fep1 occupancy. TAP-Fep1 binding to the *str2^+^* (positions −80 to −49) and *str1^+^* (positions −873 to −809) promoter regions was calculated by measuring the enrichment of specific amplified *str2^+^* and *str1^+^* promoter fragments relative to an 18S ribosomal DNA coding region. ChIP data were calculated as values of the largest amount of chromatin measured (fold enrichment). Results are shown as averages ± SD from three independent experiments, each performed in biological triplicate. Asterisks indicate statistical significance (****p < 0.0001, one-way ANOVA with Dunnett’s multiple comparisons test, comparing iron-replete cells expressing TAP-Fep1).

To further investigate whether Fep1 physically occupies the *str2^+^* and *str1^+^* promoters in an iron-dependent manner, we used a *fep1Δ php4Δ* mutant strain expressing either an untagged or TAP-tagged *fep1^+^* allele, which had been re-integrated. Since this mutant strain lacks Php4, it ensures that the transcription of the re-integrated *fep1^+^* or *TAP-fep1^+^* alleles occurs independently of Php4, allowing their constitutive expression regardless of cellular iron levels (Mercier et al., 2008; Jbel et al., 2009; Mercier and Labbé, 2009). Thus, this biological system enabled us to separate the iron-dependent DNA binding activity of Fep1 and TAP-Fep1 from potential changes in their gene expression. Under this setup context, we used a ChIP approach to test whether the presence of TAP-Fep1 could be detected at the *str2^+^* and *str1^+^* promoters *in vivo* (Figure 2A). We probed for promoter occupancy using primers specific to the *str2^+^* and *str1^+^* promoter regions, which are known to contain GATA elements that are bound by Fep1 *in vitro* (Pelletier et al., 2003). ChIP analysis revealed that TAP-Fep1 occupied the *str2^+^* and *str1^+^* promoters at maximum levels when cells were treated with FeCl_3_ (100 μM). Under these conditions, TAP-Fep1 occupancy at the *str2^+^* and *str1^+^* promoters increased 7.9- and 10.7-fold, respectively, compared to a control region encoding the 18S ribosomal RNA (Figure 2B). In contrast, when *fep1Δ php4Δ* cells expressing TAP-Fep1 were treated with Dip (250 μM), only low levels of *str2^+^* and *str1^+^* promoter fragments were immunoprecipitated (Figure 2B). In iron-replete cells, TAP-Fep1 exhibited 8.8- and 13.4-fold higher binding to the *str2^+^* and *str1^+^* promoter regions, respectively, compared to iron-starved cells expressing TAP-Fep1 (Figure 2B). As negative controls, untagged Fep1 immunoprecipitated only background levels of the *str2^+^* and *str1^+^* promoter regions (Figure 2B). Collectively, these results showed that TAP-Fep1 is recruited to *str2^+^* and *str1^+^* promoters under conditions of high levels of iron.

### Str2 localizes to the vacuolar membrane

**Figure 3:**
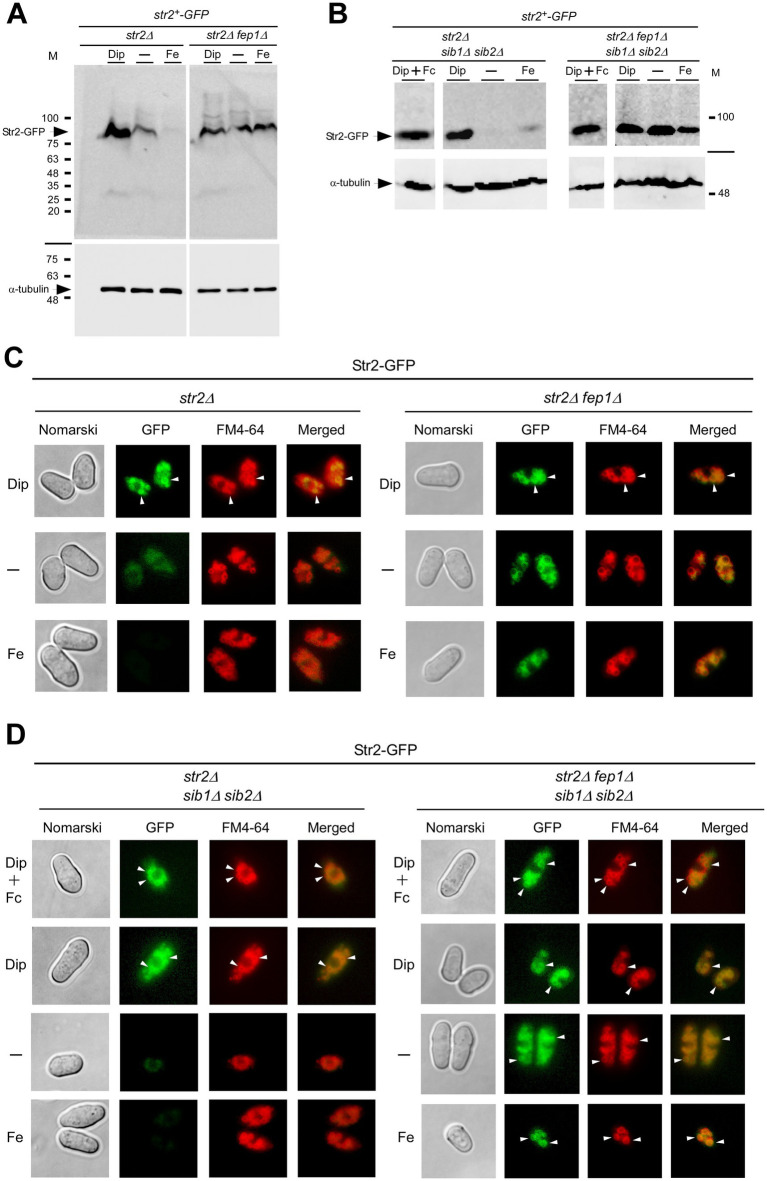
Effect of fep1Δ deletion on Str2 protein expression and localization. **(A,B)** *str2Δ*, *str2Δ fep1Δ*, *str2Δ sib1Δ sib2Δ*, and *str2Δ fep1Δ sib1Δ sib2Δ* strains expressing Str2-GFP were grown to an OD_600_ of 1.0. The cultures were then either left untreated (−) or treated with Dip (250 μM) or FeCl_3_ (Fe, 100 μM) for 3 h. In the case of *str2Δ sib1Δ sib2Δ* and *str2Δ fep1Δ sib1Δ sib2Δ* strains, Dip-treated cells were either left without further supplementation or supplemented with holo-Fc (1 μM) during the final hour of treatment. Whole cell extracts were analyzed by immunoblot assays with anti-GFP and anti-*α*-tubulin antibodies. The positions of molecular weight markers (in kDa) are indicated on the right side. **(C,D)** Fluorescence microscopy was performed on cells incubated from each group of cultures described in *panels A* and *B* to visualize the localization of Str2-GFP (*center left*). Cell morphology was examined using Nomarski optics (*far left*). White arrowheads point to examples of vacuole membranes. FM4-64 staining (*center right*), a marker of vacuolar membranes, was also visualized by fluorescence microscopy. Merged images of Str2-GFP and FM4-64 are shown in the *far-right* panels. The microscopy results are representative of three independent experiments, each performed in biological triplicate.

The iron- and Fep1-dependent regulation of *str2^+^* expression led us to investigate whether the steady-state protein levels of Str2 mirrored the changes in *str2^+^* transcript levels as a function of iron availability. A functional *str2^+^-GFP* allele, expressed under the control of its own promoter, was integrated into the genomes of *str2Δ*, *str2Δ fep1Δ*, *str2Δ sib1Δ sib2Δ*, and *str2Δ fep1Δ sib1Δ sib2Δ* mutant strains. The first two strains were grown to an OD_600_ of 1.0 and either left untreated or treated with Dip (250 μM) or FeCl_3_ (100 μM) for 3 h. The last two strains, which are deficient in Fc biosynthesis, were grown under same conditions as the first two. However, when they reached an OD_600_ of 1.0, Dip-treated cells were either incubated without Fc supplementation or supplemented with holo-Fc (1 μM) during the final hour of treatment. Whole cell extracts were then prepared and analyzed by immunoblotting. The results showed that Str2-GFP steady-state levels correlated with *str2^+^* transcript levels, increasing in the presence of Dip but remaining low in untreated or iron-treated *str2Δ* and *str2Δ sib1Δ sib2Δ* cells harboring a *str2^+^-GFP* allele (Figures 3A,B). In contrast, in *str2Δ fep1Δ* and *str2Δ fep1Δ sib1Δ sib2Δ* cells expressing *str2^+^-GFP*, the steady-state levels of Str2-GFP remained higher in both untreated and iron-treated conditions compared to strains with a functional *fep1^+^* allele (Figures 3A,B). Furthermore, steady-state levels of Str2-GFP were detected when *str2Δ sib1Δ sib2Δ* and *str2Δ fep1Δ sib1Δ sib2Δ* strains harboring an integrated *str2^+^-GFP* allele were incubated in the presence of Dip with Fc supplementation (1 μM) (Figure 3B).

Next, we aimed to determine the subcellular localization of Str2-GFP when expressed in untreated, iron-replete, and iron-starved *str2Δ*, *str2Δ fep1Δ*, *str2Δ sib1Δ sib2Δ*, and *str2Δ fep1Δ sib1Δ sib2Δ* strains. Moreover, we examined the Str2-GFP fluorescent signal in *str2Δ sib1Δ sib2Δ* and *str2Δ fep1Δ sib1Δ sib2Δ* strains that had been incubated with exogenous Fc (1 μM) under low-iron conditions. Fluorescence microscopy analysis of iron-starved *str2Δ* and *str2Δ sib1Δ sib2Δ* cells expressing Str2-GFP revealed that Str2-GFP-mediated fluorescence was localized to the vacuole membranes, regardless of the presence of exogenous Fc in the case of *str2Δ sib1Δ sib2Δ* cells (Figures 3C,D). The Str2-GFP signal colocalized with the vacuole-staining dye FM4-64, which served as a marker for the vacuolar membrane (Figures 3C,D). Consistent with iron-dependent repression of *str2^+^* expression, Str2-GFP fluorescence levels were markedly reduced in Str2-GFP-expressing *str2Δ* and *str2Δ sib1Δ sib2Δ* cells grown under basal or high-iron conditions (Figures 3C,D). In contrast, the fluorescence signal at the vacuole membrane persisted when GFP-tagged *str2^+^* was expressed in *str2Δ fep1Δ* and *str2Δ fep1Δ sib1Δ sib2Δ* strains under all tested conditions (Figures 3C,D). Taken together, these results led us to conclude that Str2 functions at the vacuole membrane in iron-starved cells, regardless of whether they are Fc prototrophic or auxotrophic. Furthermore, the vacuolar localization of Str2 remains unchanged in the presence of exogenous Fc.

### Iron accumulates in vacuoles from Str2-expressing cells

**Figure 4:**
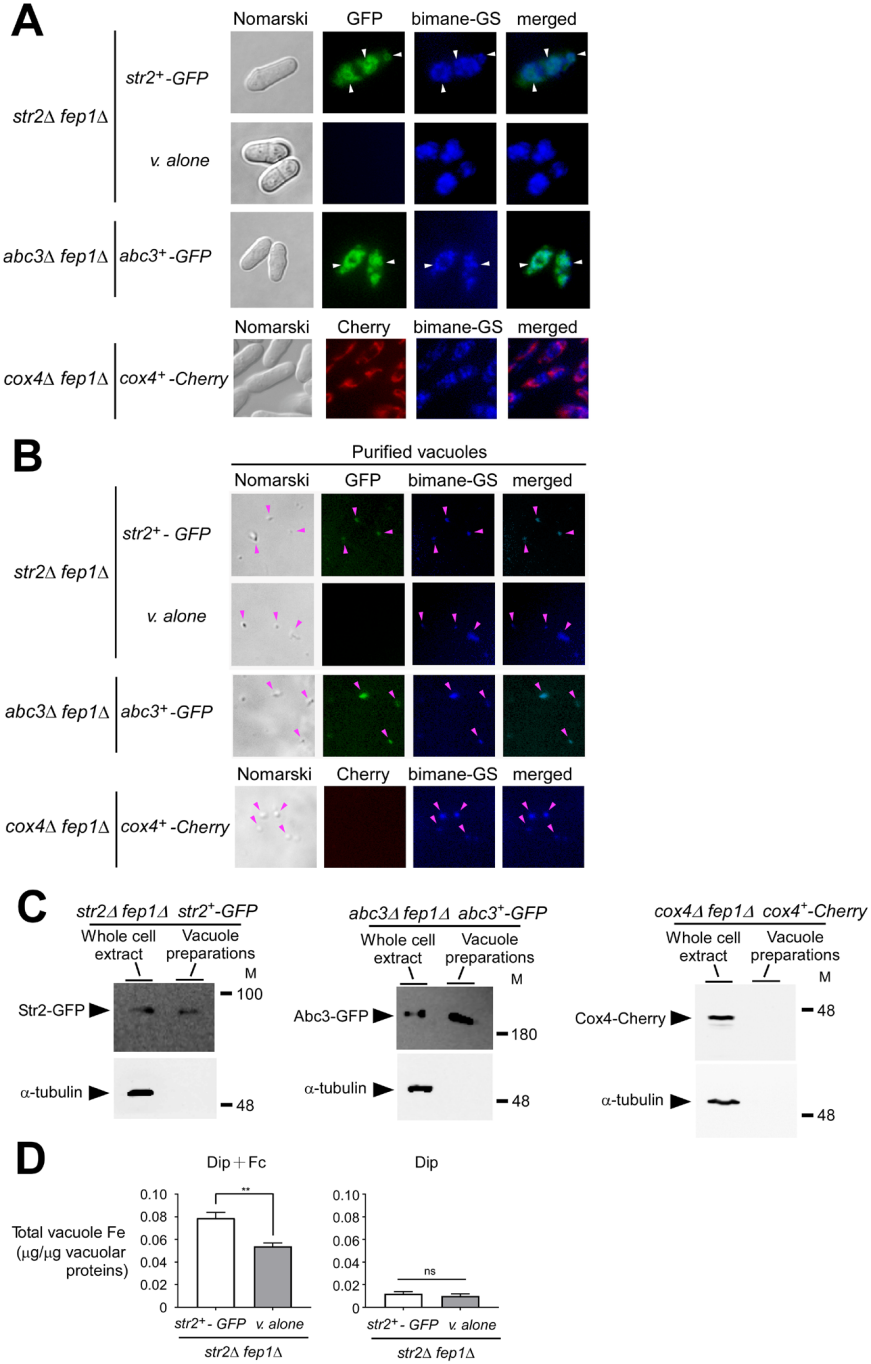
Str2 co-purifies with yeast vacuoles that exhibit iron accumulation. **(A)** *str2Δ fep1Δ* cells expressing either an empty plasmid (v. alone) or the *str2^+^-GFP* allele were grown in YES medium to an OD_600_ of 1.0. The cultures were then incubated with Dip (250 μM) for 3 h. In the final hour of this treatment, holo-Fc (1 μM) was added, followed by vacuole isolation from each group of cultures. For the *abc3Δ fep1Δ* and *cox4Δ fep1Δ* mutant strains, the *abc3^+^-GFP* and *cox4^+^-Cherry* alleles were reintroduced, and these cells were cultured under the same conditions as the *str2Δ fep1Δ* cells expressing GFP-tagged Str2. The above-mentioned cultures were examined by fluorescence microscopy to visualize the cellular localization of Str2-GFP, Abc3-GFP, and Cox4-Cherry (*center left*), along with the accumulation of fluorescent bimane-GS (*center right*). Merged images of GFP or Cherry and bimane-GS fluorescent signals are shown in the *far-right panels*. Cell morphology was examined using Nomarski optics (*far left*). White arrowheads indicate examples of vacuole membranes. **(B)** Vacuoles were purified from each group of cultures described in *panel A* and visualized by fluorescence microscopy to observe Str2-GFP, Abc3-GFP, Cox4-Cherry, and bimane-GS fluorescent signals. Merged images of GFP and bimane-GS fluorescent signals are displayed in the *far-right panels*. Pink arrowheads point to examples of purified vacuoles. **(C)** Aliquots of total cell extracts and vacuole preparations from each group of cultures were analyzed by immunoblotting using anti-GFP, anti-Cherry, and anti-α-tubulin antibodies. Abc3-GFP was used as a known vacuolar membrane marker, whereas the absence of the Cox4-Cherry and α-tubulin signals confirmed the specificity of the vacuole preparations. **(D)** Purified vacuoles from *str2Δ fep1Δ* cells expressing an empty plasmid (v. alone) or the *str2^+^-GFP* allele were analyzed using a BPS-based spectrophotometric method to quantitatively measure iron levels. Cells were incubated with Dip (250 μM) for 3 h. In the final hour of this treatment, holo-Fc (1 μM) was added or omitted. Results are representative of three independent experiments. Data are presented as mean ± SD. Statistical significance is indicated by asterisks, with **p < 0.01 (determined by one-way ANOVA with Dunnett’s multiple comparisons test, comparing against cells expressing Str2-GFP).

When the *fep1^+^* gene is disrupted, *fep1* null strains exhibit substantially increased expression of several genes encoding proteins involved in cellular iron homeostasis, such as iron transporters, iron–sulfur proteins, and iron-consuming proteins (Rustici et al., 2007; Brault et al., 2015). Based on these previous findings, we used *str2Δ fep1Δ* and *abc3Δ fep1Δ* mutant strains expressing re-integrated *str2^+^-GFP* and *abc3^+^-GFP* alleles, respectively. Moreover, for the *str2Δ fep1Δ* strain, an integrative empty vector was transformed as a negative control. Cells were grown to an OD_600_ of 1.0 and then incubated with Dip (250 μM) for 3 h. During the final hour of treatment, holo-Fc (1 μM) was added, and aliquots of the cells were subsequently visualized using fluorescence microscopy. The results showed that Str2-GFP-associated fluorescence was detected at the vacuolar membrane (Figure 4A). To further validate the vacuolar localization of Str2-GFP, the vacuolar-sequestered fluorescent compound bimane-GS was used as a marker (Sarry et al., 2007). Merged images showed that Str2-GFP and bimane-GS shared a similar subcellular localization pattern within the cells (Figure 4A). As an additional control, we examined the vacuolar localization of Abc3-GFP, a known vacuolar membrane transporter (Pouliot et al., 2010). Under identical growth conditions, Abc3-GFP fluorescence was observed at the vacuoles and co-localized with the bimane-GS marker (Figure 4A). In contrast, no green fluorescence signal was detected in *str2Δ fep1Δ* cells containing an empty vector (Figure 4A). To distinguish protein localization at the vacuole from other organelles, we used *cox4Δ fep1Δ* cells expressing Cox4-Cherry, a protein known to be a mitochondrial resident marker. Results showed that the fluorescence associated with Cox4-Cherry exhibited a distinct localization pattern compared to the subcellular localization of Str2-GFP and showed no overlap with the bimane-GS signal (Figure 4A).

Vacuoles were purified from the above-mentioned cultures, and sample aliquots were examined by fluorescence microscopy. The results indicated that the vacuoles maintained their integrity throughout the purification process, as the bimane-GS-associated blue fluorescence was retained in most of the isolated vacuoles (Figure 4B). Furthermore, some of these isolated vacuoles exhibited a green fluorescence signal when purified from *str2Δ fep1Δ* and *abc3Δ fep1Δ* cells expressing *str2^+^-GFP* and *abc3^+^-GFP*, respectively (Figure 4B). In contrast, vacuoles isolated from *str2Δ fep1Δ* cells containing an empty vector or *cox4Δ fep1Δ* cells expressing Cox4-Cherry lacked any green and red fluorescence signal, respectively.

Proteins were extracted from vacuole preparations and analyzed by immunoblot assays. Str2-GFP was detected as vacuolar protein when yeast vacuoles were isolated from *str2Δ fep1Δ* cells expressing *str2^+^-GFP* (Figure 4C). Similarly, Abc3-GFP, a known vacuolar membrane protein, was detected when vacuolar proteins were analyzed by immunoblotting from Abc3GFP-expressing *abc3Δ fep1Δ* cells (Figure 4C). In contrast, when proteins were extracted from vacuole preparations, immunoblot experiments consistently showed no signal corresponding to Cox4-Cherry.

Vacuoles isolated from *str2Δ fep1Δ* cells expressing *str2^+^-GFP* or containing an empty plasmid were analyzed for their iron content by a BPS-based spectrophotometric method (Rad et al., 2007; Pouliot et al., 2010). In *str2Δ fep1Δ* cells treated with Fc and expressing *str2^+^-GFP*, purified vacuoles exhibited a total iron concentration of 0.079 μg per μg of vacuolar proteins (Figure 4D). In contrast, vacuoles from *str2Δ fep1Δ* cells harboring an empty plasmid showed 31.7% less iron (0.054 μg iron/μg of vacuolar proteins) compared to those isolated from Str2GFP-expressing *str2Δ fep1Δ* cells (Figure 4D). As a control, vacuoles isolated from *str2Δ fep1Δ* cells expressing *str2^+^-GFP* or carrying an empty plasmid, and incubated with Dip without Fc supplementation, displayed very low total iron concentrations (0.012 and 0.010 μg iron/μg of vacuolar proteins, respectively) (Figure 4D). Interestingly, these results showed that adding Fc in the absence of Str2-GFP led to an increase in vacuolar iron concentration, although not to the same extent as when Str2-GFP was expressed. This observation suggests the possible existence of an alternative transport system that facilitates iron transport to the vacuole, independent of Str2. Nonetheless, these findings strongly suggested that Str2 plays a role in mobilizing iron within the vacuole when cells are grown with holo-Fc as the sole source of iron.

### The loss of Str2 function in sib1Δ sib2Δ mutant cells leads to growth defect on iron-poor media containing Fc

**Figure 5:**
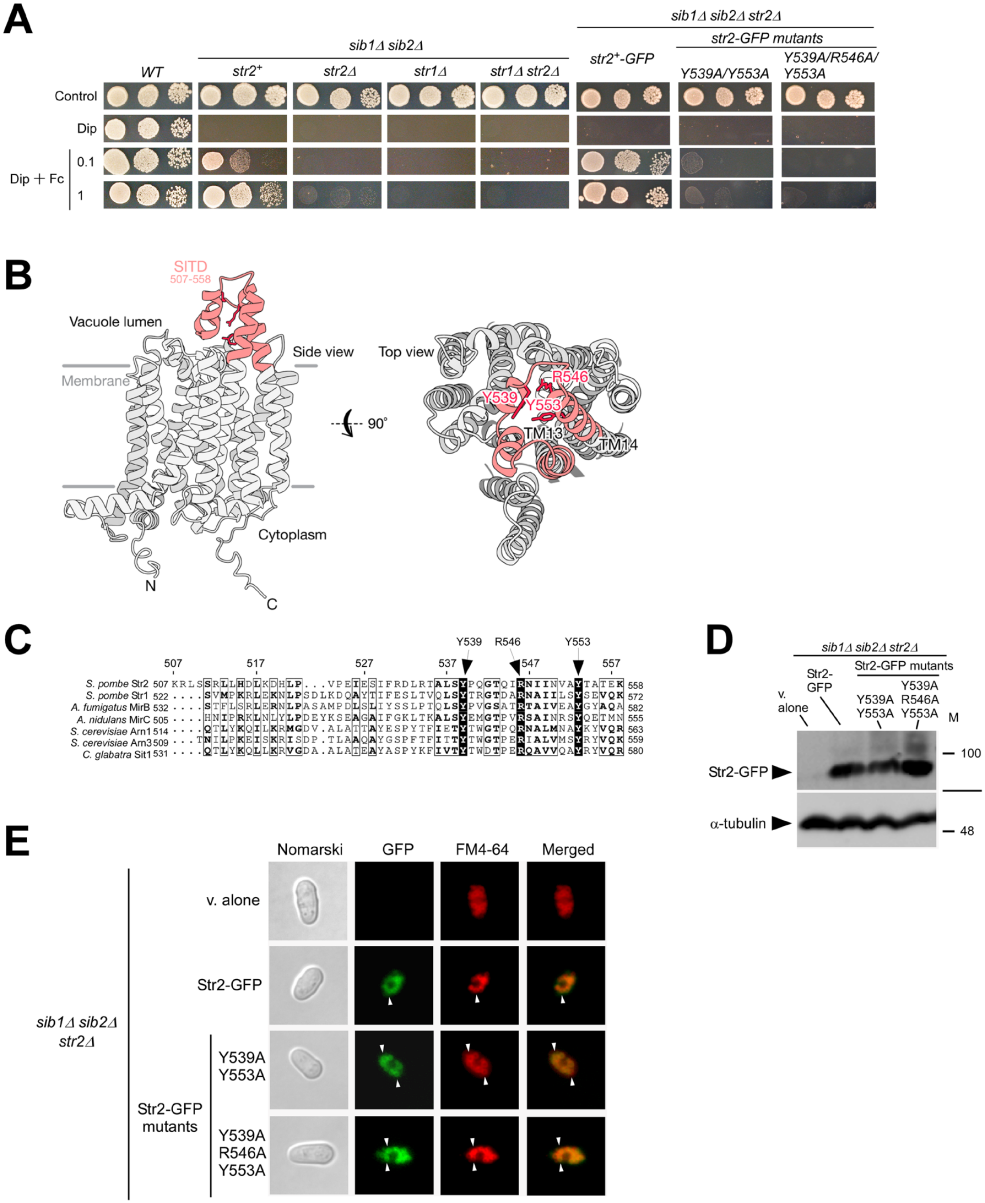
*sib1Δ sib2Δ* mutant cells require Str2 for Fc-dependent growth. **(A)** Wild-type (WT), *sib1Δ sib2Δ*, *sib1Δ sib2Δ str2Δ*, *sib1Δ sib2Δ str1Δ*, and *sib1Δ sib2Δ str1Δ str2Δ* cells, as well as *sib1Δ sib2Δ str2Δ* cells expressing *str2^+^-GFP*, *str2-Y539A/Y553A-GFP* or *str2-Y539A/R546A/Y553A-GFP* alleles were grown in YES medium to an OD_600_ of 1.0, and then spotted in serial dilutions (6,000 cells/10 μL; 600 cells/10 μL; and, 60 cells/10 μL) onto medium without Dip or Fc supplementation (control) or supplemented with Dip (140 μM) or a combination of Dip and Fc (0.1 or 1 μM). **(B)** A predicted three-dimensional structure of Str2 is shown, with potential transmembrane-spanning domains indicated in gray and the STID highlighted in red. **(C)** Amino acid alignment of the predicted carboxyl-terminal final loop of *S. pombe* Str2 with other predicted final loops found in *S. pombe* Str1, *A. fumigatus* MirB, *A. nidulans* MirC, *C. glabrata* Sit1, and *S. cerevisiae* Arn1 and Arn3. Arrows indicate three highly conserved Tyr (Y) and Arg (R) residues. Amino acid sequence numbers refer to their position relative to the first amino acid of each protein. **(D)** Whole extracts from aliquots of iron-starved *sib1Δ sib2Δ str2Δ* cells expressing an empty plasmid (v. alone), *str2^+^-GFP*, *str2-Y539A/Y553A-GFP* or *str2-Y539A/R546A/Y553A-GFP* alleles were analyzed by immunoblotting using anti-GFP and anti-α-tubulin antibodies. The positions of molecular weight markers (in kDa) are indicated on the right side. **(E)** *sib1Δ sib2Δ str2Δ* cells expressing an empty plasmid (v. alone), *str2^+^-GFP*, *str2-Y539A/Y553A-GFP* or *str2-Y539A/R546A/Y553A-GFP* alleles treated with Dip were analyzed by fluorescence microscopy to detect GFP fluorescence (*center left*), along with FM4-64 staining (*center right*). Merged images of GFP and FM4-64 signals are shown in the *far-right panels*. Cell morphology was examined using Nomarski optics (*far left*). White arrowheads indicate examples of vacuole membranes.

The Fc biosynthetic pathway is essential for *S. pombe* survival under iron-limiting conditions (Mercier and Labbé, 2010; Brault et al., 2022). The *sib1^+^* and *sib2^+^* genes are necessary for Fc production in *S. pombe* (Schrettl et al., 2004b; Mercier and Labbé, 2010; Brault et al., 2022). Yeast strains with deletions of these two genes (*sib1Δ sib2Δ*) are unable to grow on iron-poor media supplemented with Dip (Figure 5A; Mercier and Labbé, 2010; Brault et al., 2022). Notably, this growth defect due to iron deficiency was rescued by adding exogenous Fc (0.1 and 1 μM) to the medium (Figure 5A). To determine whether Str2 is required for the utilization of exogenous Fc, the *str2^+^* gene was disrupted in the *sib1Δ sib2Δ* strain and tested on Dip-supplemented media containing 0.1 and 1 μM Fc. As shown in Figure 5A, *sib1Δ sib2Δ str2Δ* mutant cells exhibited a severe growth defect on this Fc-supplemented medium compared to wild-type and *sib1Δ sib2Δ* cells expressing *str2^+^* (Figure 5A). Given the established role of Str1 in the uptake of exogenous Fc (Plante and Labbé, 2019; Brault et al., 2022), we validated that its inactivation (*str1Δ*) in the *sib1Δ sib2Δ* strain led to an inability to grow on Dip-supplemented media containing 0.1 and 1 μM Fc (Figure 5A). Moreover, the results consistently showed that the *sib1Δ sib2Δ str1Δ str2Δ* quadruple mutant strain was unable to grow in the presence of exogenous Fc under low-iron conditions (Figure 5A).

Amino acid sequence analysis of Str2 suggests that the protein belongs to the MFS-type transporter family (Rutherford et al., 2024). Topological models of Str2 predict the presence of 14 transmembrane spans connected by hydrophilic loops (Figure 5B). The final loop is predicted to contain a putative siderophore transporter domain (SITD) with highly conserved amino acid residues (Nevitt and Thiele, 2011). Among these conserved residues, Tyr^539^, Arg^546^, and Tyr^553^ in Str2 are found within the SITD of other predicted or known hydroxamate-type siderophore transporters, including Str1 (*S. pombe*), MirB (*Aspergillus fumigatus*), MirC (*Aspergillus nidulans*), Sit1 (*Candida glabrata*), Arn1, and Arn3 (*S. cerevisiae*) (Figure 5C; Yun et al., 2000; Kim et al., 2002; Kim et al., 2005; Philpott, 2006; Haas et al., 2008; Nevitt and Thiele, 2011; Raymond-Bouchard et al., 2012; Plante and Labbé, 2019). Based on previous studies that had demonstrated the functional importance of conserved Tyr residues in the SITD domain of *S. pombe* Str1 (Tyr^553^ and Tyr^567^) and *C. glabrata* Sit1 (Tyr^575^) (Nevitt and Thiele, 2011; Plante and Labbé, 2019), we generated two mutant derivatives of Str2. In the first mutant, Tyr^539^ and Tyr^553^ were substituted with Ala residues, whereas in the second mutant, Tyr539, Arg546, and Tyr553 were replaced by Ala residues. To assess the role of Str2 in Fc-dependent growth under iron-deficient conditions, spot assays were performed using *sib1Δ sib2Δ str2Δ* cells expressing either *str2^+^-GFP*, *str2-Y539A/Y553A-GFP*, or *str2-Y539A/R546A/Y553A-GFP* allele. As shown in Figure 5A, *sib1Δ sib2Δ str2Δ* cells expressing *str2^+^-GFP* exhibited growth on Dip-supplemented media containing 0.1 and 1 μM Fc. In contrast, *sib1Δ sib2Δ str2Δ* cells expressing *str2-Y539A/Y553A-GFP* or *str2-Y539A/R546A/Y553A-GFP* allele displayed a severe growth defect when spotted on iron-depleted medium supplemented with Fc (0.1 and 1 μM) compared to those expressing *str2^+^-GFP* (Figure 5A).

To confirm that all *GFP-tagged str2* alleles were expressed in *sib1Δ sib2Δ str2Δ* cells, steady-state protein levels of Str2-GFP and its mutant derivatives were analyzed by immunoblot assays. The results showed that all these proteins were expressed in the *sib1Δ sib2Δ str2Δ* strain (Figure 5D). As a negative control, whole cell extracts from *sib1Δ sib2Δ str2Δ* cells transformed with an empty plasmid were also analyzed by immunoblotting.

To ensure that the mutated forms, Str2-Y539A/Y553A-GFP and Str2-Y539A/R546A/Y553A-GFP, exhibited the same subcellular localization as the wild-type Str2-GFP protein, microscopic analyses were performed on the two GFP-tagged mutants alongside Str2-GFP. The results showed that Str2-GFP and its mutant derivatives displayed similar fluorescence patterns, localizing to the vacuoles and colocalizing with the vacuole-staining dye FM4-64 (Figure 5E). Taken together, the results showed that Str2 is required for sustaining cell growth in the presence of exogenous holo-Fc under iron-starvation conditions. Furthermore, the conserved amino acid residues Tyr^539^, Arg^546^, and Tyr^553^, located within the predicted final loop of Str2, are critical for its Fc-related function.

### Str2 mutants exhibit reduced iron levels in their vacuoles when Fc is used as the sole iron source

**Figure 6:**
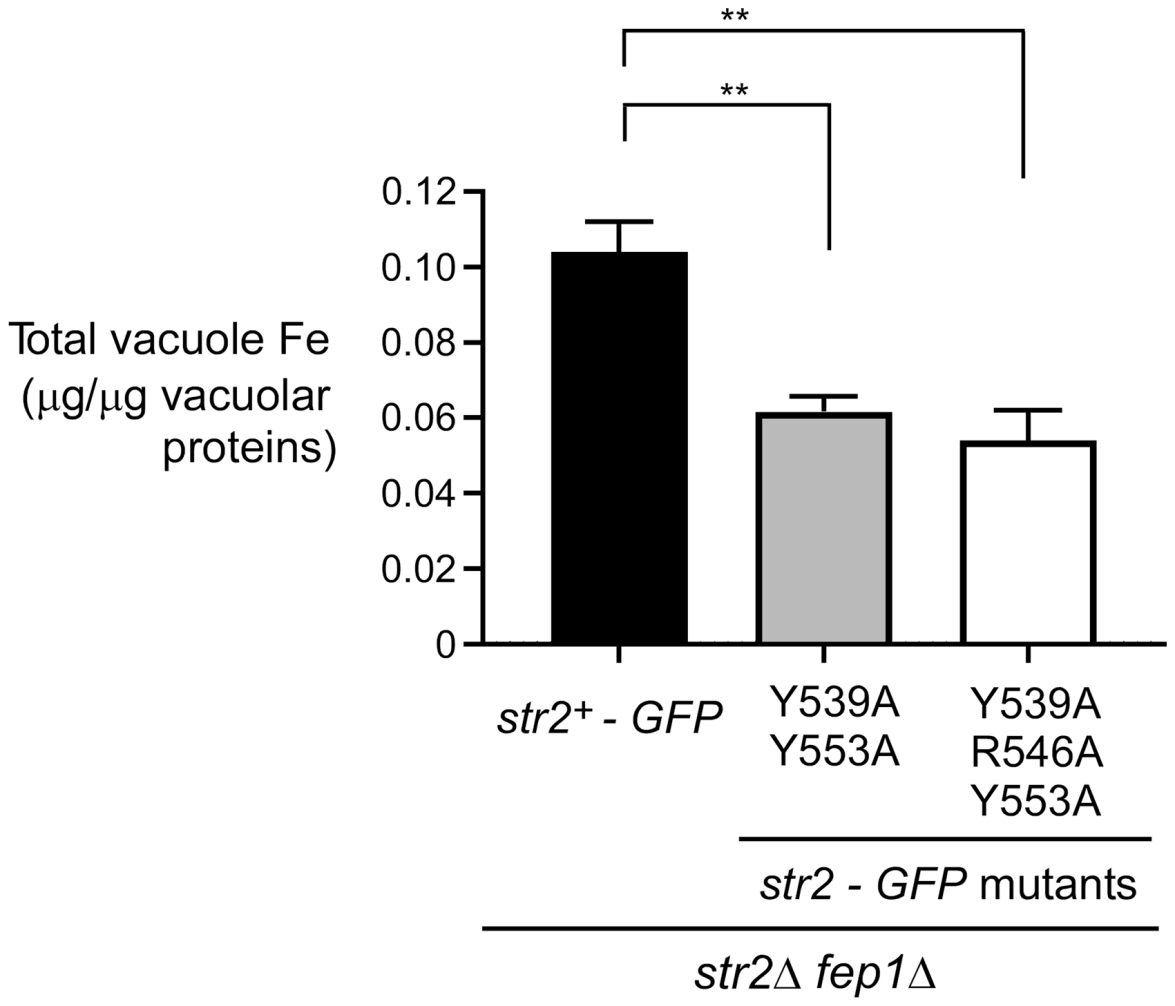
The Tyr^539^, Arg^546^, and Tyr^553^ residues of Str2 play an important role for maximal iron accumulation in vacuoles. *str2Δ fep1Δ* cells expressing *str2^+^-GFP*, *str2-Y539A/Y553A-GFP* or *str2-Y539A/R546A/Y553A-GFP* alleles were grown in YES medium to an OD_600_ of 1.0. The cultures were then incubated with Dip (250 μM) for 3 h. In the final hour of this treatment, holo-Fc (1 μM) was added, followed by vacuole isolation from each group of cultures. Purified vacuoles from each culture were analyzed using a BPS-based spectrophotometric method to quantitatively measure iron levels. Results are representative of three independent experiments. Data are presented as mean ± SD. Statistical significance is indicated by asterisks, with **p < 0.01 (determined by one-way ANOVA with Dunnett’s multiple comparisons test, comparing against cells expressing Str2-GFP).

We next assessed the impact of the Str2-Y539A/Y553A-GFP and Str2-Y539A/R546A/Y553A-GFP mutants on the ability of *str2Δ fep1Δ* cells to mobilize iron within vacuoles. Logarithmic phase *str2Δ fep1Δ* cells expressing the *str2^+^-GFP*, *str2-Y539A/Y553A-GFP*, or *str2-Y539A/R546A/Y553A-GFP* alleles were treated with Dip (250 μM) for 3 h. During the final hour of treatment, holo-Fc (1 μM) was added, followed by vacuole isolation from each culture. Purified vacuoles were analyzed using a BPS-based spectrophotometric method to quantify iron concentrations. In *str2Δ fep1Δ* cells expressing the *str2-Y539A/Y553A-GFP* and *str2-Y539A/R546A/Y553A-GFP* alleles, purified vacuoles contained 0.062 and 0.054 μg iron/μg of vacuolar proteins, respectively (Figure 6). These values represent 40.4 and 48.1% less iron compared to *str2Δ fep1Δ* cells expressing the *str2^+^-GFP* allele, which had 0.104 μg iron/μg of vacuolar proteins (Figure 6). Taken together, these results indicated that substituting the Tyr^539^, Arg^546^, and Tyr^553^ residues with alanines in Str2 lead to a reduction in vacuolar iron levels when iron-starved cells are grown with holo-Fc as the sole source of iron.

## Discussion

Unlike *Saccharomyces cerevisiae*, *S. pombe* synthesizes and secretes Fc (Schrettl et al., 2004b). Consistently, *S. pombe* possesses a cell-surface transporter, Str1, that can take up Fc from the extracellular environment (Plante and Labbé, 2019). Although *S. cerevisiae* lacks the proteins necessary to produce siderophores, it can assimilate various types of siderophores, including Fc, ferrioxamine B, triacetylfusarinine C, and enterobactin secreted by other microbes (Philpott, 2006). *S. cerevisiae* expresses four siderophore-specific transporters (Arn1, Arn2/Taf1, Arn3/Sit1, and Arn4/Enb1) that mediate the uptake of siderophore-bound iron from the environment (Lesuisse et al., 1998; Heymann et al., 1999, 2000a, 2000b; Yun et al., 2000; Philpott, 2006). Among them, Arn1 transports Fc and other hydroxamate-type siderophores (Heymann et al., 2000b; Yun et al., 2000). Recent studies have shown that Fc produced by *S. pombe* promotes the growth of Arn1-expressing *S. cerevisiae* cells when Fc is used as the sole iron source (Brault et al., 2022). Due to fundamental differences between the two yeasts, their pathways involved in Fc metabolism differ in some aspects.

In the case of *S. pombe*, to dissociate its capacity to acquire exogenous Fc from its ability to synthesize endogenous Fc, we used a strain with deletions in the *sib1^+^* and *sib2^+^* genes (*sib1Δ sib2Δ*), which blocks *de novo* Fc biosynthesis by eliminating these enzymes from the pathway. Therefore, *sib1Δ sib2Δ* cells rely solely on their cell surface Fc transporter Str1 to acquire exogenous Fc. In the present study, microscopic analyses from *str2Δ* and *str2Δ sib1Δ sib2Δ* cells expressing *str2^+^-GFP* showed that Str2-GFP fluorescent signal is primarily observed to the vacuolar membrane under low-iron conditions. Furthermore, in the case of *str2Δ sib1Δ sib2Δ* cells, the vacuolar localization of Str2-GFP remains unchanged when the cells are exposed to exogenous Fc. These findings are different from those found in the case of siderophore transporters in *S. cerevisiae* (Philpott, 2006). None of the four siderophore transporters (Arn1 to Arn4) is a permanent resident vacuolar protein in *S. cerevisiae* (Philpott, 2006). The Arn1 and Arn3 transporters are localized to the trans-Golgi network where they are sorted to endosomal secretory vesicles (Kim et al., 2002; Moore et al., 2003; Kim et al., 2005). A model posits that exogenous Fc initially enters the cell through fluid-phase endocytosis, where it encounters Arn1 in the early endosome. Binding of Fc to Arn1 triggers its relocalization from the endosome to the plasma membrane. Once at the plasma membrane, extracellular Fc can bind Arn1, promoting its ubiquitination, internalization, and cycling between the plasma membrane and endosomes, while mediating the transport of Fc into the cell (Philpott, 2006). In contrast, when siderophores are no longer available outside the cell, the Arn1, Arn2, and Arn3 transporters located in the late Golgi network are sorted to the vacuole for degradation. In the case of Arn4, however, it exhibits a distinct trafficking pattern, being directed to the cell surface even in the absence of its siderophore, enterobactin (Philpott and Protchenko, 2008).

Our results showed that *S. pombe* cells deficient in Fc biosynthesis (*sib1Δ sib2Δ*) require *str2^+^* for Fc-dependent growth under low-iron conditions. Since Str2 localizes to the vacuolar membrane, this suggests that, after Fc uptake through the plasma membrane by Str1, it must be delivered to Str2 at the vacuole. In the case of *S. pombe* Str1, it is unclear whether this siderophore transporter undergoes intracellular trafficking upon Fc binding. In *S. cerevisiae*, Fc binding to Arn1 triggers its internalization. In the endosome, Fc bound to Arn1 is thought to be translocated into the cytosol, where iron is released from the siderophore likely through degradation of Fc (Philpott, 2006). One possibility is that a similar Fc-mediated internalization of Str1 occurs in *S. pombe*. However, this would require that Fc maintains its integrity after its translocation into the cytosol, allowing it to subsequently bind to Str2 and be transported into the vacuole, where iron would then be dissociated from the siderophore.

Studies in budding and fission yeasts have shown that the vacuole serves as a storage compartment for metal ions, either to detoxify the cell or to act as a reservoir, enabling cell growth under conditions of metal ion deficiency (Ramsay and Gadd, 1997; Bellemare et al., 2002; Rees et al., 2004; Simm et al., 2007; Singh et al., 2007). In *S. cerevisiae*, different proteins are involved in the mobilization of vacuolar iron stores. Under iron-replete conditions, the vacuolar iron transporter Ccc1 transfers iron from the cytosol to the vacuole (Li et al., 2001). Conversely, in cells undergoing a transition from high to low iron levels, the vacuolar iron transporter Smf3 mobilizes stored iron from the vacuole to the cytosol (Portnoy et al., 2000). Copper-and iron-deficient cells also activate the expression of Fre6, a cupric/ferric reductase found on the vacuolar membrane that reduces vacuolar Cu^2+^ and Fe^3+^ ions (Rees and Thiele, 2007; Singh et al., 2007). The reduced iron (Fe^2+^) is then transported out of the vacuole by the Fet5/Fth1 oxidase/permease heteromeric complex (Urbanowski and Piper, 1999). In *S. pombe*, the mechanism of vacuolar iron mobilization in response to iron deficiency is not well understood and may differ due to the absence of orthologs for Smf3, Fre6, Fth1, and Fet5. *S. pombe* has a single Ccc1-like protein, Pcl1, which is thought to mediate vacuolar iron storage. Deletion of the *pcl1^+^* gene (*pcl1Δ*) results in a mutant strain with reduced cellular iron content compared to the wild-type strain (Pouliot et al., 2010). However, the definitive role of Pcl1 in vacuolar iron storage has yet to be confirmed.

In this study, vacuolar iron concentration is lower in *str2Δ fep1Δ* cells lacking Str2 compared to control *str2Δ fep1Δ* cells expressing a functional *str2^+^-GFP* allele. The iron appears to be in an inorganic form, dissociated from Fc within the vacuole, as we were unable to detect holo-Fc in intact chelated form. MFS-type transporters contain two bundles of six or seven membrane-spanning alpha-helices. These two bundles come together to form a central pore, and the transporters operate via an alternating-access mechanism. This mechanism involves a rocker-switch-like movement, described as alternating between outward-open and inward-open conformations, triggered by substrate binding. Notably, the last two transmembrane domains and the final hydrophilic loop are unique to MFS-type fungal siderophore-iron transporters and are not shared by other related MFS transporters (Kim et al., 2005). Considering this, it is plausible that the SITD region of Str2, located in the final predicted loop on the luminal side of the vacuole, plays a critical role in facilitating the conformational change to the inward-open state. Fc may sequentially enter through the rocker-switch-like movement and subsequently bind to the C-terminal Tyr-Arg-Tyr residues of the Str2 SITD domain. In this way, the SITD region likely attracts Fc, forming a sink for Fc to bind to, before iron is extracted from Fc by an unknown mechanism and stored in the vacuole.

Iron accumulation in vacuoles when holo-Fc is available to Str2-GFP-expressing *str2Δ fep1Δ* cells is specific to Str2-GFP, as isogenic cells expressing *str2-Y539A/Y553A-GFP* and *str2-Y539A/R546A/Y553A-GFP* mutant alleles exhibited a decrease in vacuolar iron content. Based on a primary sequence alignment of Str2 with other fungal MFS-type siderophore-iron transporters, Tyr^539^, Arg^546^, and Tyr^553^ residues were mutated, as they were predicted to be located in a conserved loop that encompasses a putative siderophore transporter domain (SITD). For example, the SITD in the Fc importer Sit1 in *C. glabrata* contains a Tyr^575^ residue corresponding to Tyr^553^ in *S. pombe* Str2. Substituting Tyr^575^ with Ala in Sit1 significantly reduces the ability of *C. glabrata* to use exogenous Fc as an iron source for growth (Nevitt and Thiele, 2011). Similarly, in *A. fumigatus* (strain ATCC 13073), the siderophore transporter MirB contains a conserved Tyr^577^ residue corresponding to Tyr^553^ in *S. pombe* Str2. Substitution of Tyr^577^ with Ala in MirB resulted in a dramatic loss of siderophore transport activity (Raymond-Bouchard et al., 2012). The last extracellular loop of the *S. cerevisiae* Fc transporter Arn1 has undergone comprehensive mutagenesis (Kim et al., 2005). Alanine substitutions of Phe^540^ and Tyr^544^ (where Tyr^544^ corresponds to Tyr^539^ in Str2) resulted in a complete loss of low-affinity Fc binding, with a significant reduction in Fc uptake activity in cells expressing the mutant F540A/Y544A allele (Kim et al., 2005). Moreover, when Gln^550^, Arg^551^, Tyr^558^, and Arg^563^ were replaced with alanines (where Arg^551^ and Tyr^558^ in *S. cerevisiae* Arn1 correspond to Arg^546^ and Tyr^553^ in *S. pombe* Str2), there was a dramatic defect in Fc binding along with a complete loss of Fc transport and Fc-dependent growth in cells expressing this mutant allele (Kim et al., 2005). Our previous studies on the *S. pombe* MFS-type transporter Str1 revealed the critical importance of two conserved Tyr residues in the last predicted loop of this Fc importer (Plante and Labbé, 2019). Conserved Tyr^553^ and Tyr^567^ in Str1 correspond to Tyr^539^ and Tyr^553^ in Str2. Fungal spores expressing a mutant version of Str1, in which residues Tyr^553^ and Tyr^567^ were replaced with alanines, were unable to complete the outgrowth phase in a timely manner compared to control spores expressing wild-type Str1 in the presence of exogenous Fc (Plante and Labbé, 2019). Together, findings from previous studies and the present work underscore the necessity of conserved Tyr residues within the last loop of MFS-type fungal siderophore-iron transporters for transporting environmental Fc into cells or other subcellular targets. Based on the extended amino acid sequence similarity between Str2 and Str1, particularly within the regions encompassing the predicted transmembrane domains, it would be interesting to identify which motif of the intracellular Fc transporter Str2 is required for its sorting to the vacuolar membrane, in contrast to Str1, which is sorted to the plasma membrane to transport Fc into the cell.

## Data Availability

The raw data supporting the conclusions of this article will be made available by the authors, without undue reservation.
